# Adoption of Mobile Health Apps in Dietetic Practice: Case Study of Diyetkolik

**DOI:** 10.2196/16911

**Published:** 2020-10-02

**Authors:** Gorkem Akdur, Mehmet Nafiz Aydin, Gizdem Akdur

**Affiliations:** 1 Department of Management Information Systems Kadir Has University Istanbul Turkey; 2 Centre for Research in Public Health and Community Care University of Hertfordshire Hatfield United Kingdom

**Keywords:** mHealth, technology acceptance, user acceptance, mobile apps, diet apps, Technology Acceptance Model, TAM, dietetics

## Abstract

**Background:**

Dietetics mobile health apps provide lifestyle tracking and support on demand. Mobile health has become a new trend for health service providers through which they have been shifting their services from clinical consultations to online apps. These apps usually offer basic features at no cost and charge a premium for advanced features. Although diet apps are now more common and have a larger user base, in general, there is a gap in literature addressing why users intend to use diet apps. We used Diyetkolik, Turkey’s most widely used online dietetics platform for 7 years, as a case study to understand the behavioral intentions of users.

**Objective:**

The aim of this study was to investigate the factors that influence the behavioral intentions of users to adopt and use mobile health apps. We used the Technology Acceptance Model and extended it by exploring other factors such as price-value, perceived risk, and trust factors in order to assess the technology acceptance of users.

**Methods:**

We conducted quantitative research on the Diyetkolik app users by using random sampling. Valid data samples gathered from 658 app users were analyzed statistically by applying structural equation modeling.

**Results:**

Statistical findings suggested that perceived usefulness (*P*<.001), perceived ease of use (*P*<.001), trust (*P*<.001), and price-value (*P*<.001) had significant relationships with behavioral intention to use. However, no relationship between perceived risk and behavioral intention was found (*P*=.99). Additionally, there was no statistical significance for age (*P*=.09), gender (*P*=.98), or previous app use experience (*P*=.14) on the intention to use the app.

**Conclusions:**

This research is an invaluable addition to Technology Acceptance Model literature. The results indicated that 2 external factors (trust and price-value) in addition to Technology Acceptance Model factors showed statistical relevance with behavioral intention to use and improved our understanding of user acceptance of a mobile health app. The third external factor (perceived risk) did not show any statistical relevance regarding behavioral intention to use. Most users of the Diyetkolik dietetics app were hesitant in purchasing dietitian services online. Users should be frequently reassured about the security of the platform and the authenticity of the platform’s dietitians to ensure that users’ interactions with the dietitians are based on trust for the platform and the brand.

## Introduction

### Background

The advancement of information and communication technologies has urged the health care industry to develop new solutions to bring better health functions to individuals by reducing costs and time as well as enhancing convenience for both service providers and users [1]. Over the last decade, considerable health services have been partially or wholly shifted to mobile phones. Since the concept of mobile health (mHealth) was first coined in 2005 [2,3], mHealth has incorporated innovative medical services, such as mobile dietetics services. Most mHealth services consist of innovative solutions such as web-based consultation systems with physicians, web-based health conferences, health-relevant data, medical inspection results via portable and wearable gadgets, and smartphone-based apps [3].

Diet apps are a subset of mHealth and typically offer services including physical activity, calorie, water intake, and weight tracking; weight goal setting; meal recipes; and meal planning [4]. Due to the excessive number of diet apps that promote physical health and well-being, it is crucial to determine whether these apps are valid and what they provide for their users [5]. Generally, users pay attention to the number of downloads, average ratings, and reviews before downloading diet apps [6]. An important feature of Diyetkolik is its dietician consultation service. Diyetkolik allows dietitians to join the platform as experts and to offer their customized services to users.

Diyetkolik was founded in 2011, and as of 2020, it has a base of around 1.5 million users [7]. It is Turkey's most widely used online dietetics platform [8]. The platform has a large database of food that is specific to Turkey. Diyetkolik has features such as calorie checker, calorie tracking, water tracking, exercise tracking, content, calculations, and reports. Users are offered 3 types of packages: users with a basic (free) membership can access Diyetkolik’s food database, track calorie intake, and read blog posts; users with a standard diet package are assigned noncustomized diet plans and can ask a limited number of questions to a dietitian; and users with a premium diet package can benefit from customized diet plans that are prepared by dietitians. Dietitians can create expert accounts on Diyetkolik, publish nutrition-related blog posts, and connect with clients who have subscribed to a premium membership. The business strategy of Diyetkolik is business to business to consumer, which is a novel approach in dietitian services in Turkey [9].

Previous reviews [10] have found that the use of nutrition and diet mHealth apps can change nutrition health behavior and improve diet. According to Carter et al [11], app users show greater adherence, retention, and weight loss compared to those shown by other nutrition and diet site users. Yet, what is unclear is why people choose to use mHealth apps and how they use them [12]. Generally, users choose any specific mobile app based on such factors as reliability, ease of use, quality, usefulness, aesthetics, trust, and recommendations by others [13]. Hence, engineers, managers, and app developers must acknowledge what end users demand. Many previous studies [14-19] have focused on mHealth apps, but there are only a few studies [20-24] that identify diet mHealth apps from the user’s perspective. The objective of this research was to analyze the influencing factors for user acceptance of a diet mHealth app and investigate and test the importance of external constructs affecting users’ behavioral intention to use. Only a few studies [3,25-27] have analyzed end-user perspectives on diet app acceptance in non-European Union countries; this study offers additional insights on mHealth user acceptance in Turkey.

### Theoretical Framework and Research Hypotheses

One of the major influential models that has been used to assess the acceptance of technologies is the Technology Acceptance Model (TAM) [28], originally developed by Davis [29] in 1989. Davis [30] has stated that regulating circumstances to obtain and adopt any technology are based on some presumptions, and the major key factors are perceived ease of use and perceived usefulness [30]. According to McFarland and Hamilton [31], extended models can increase the explanation and forecasting of acceptance. Considering innovative technologies and their effects, Thompson et al [32] discussed that perceived ease of use and perceived usefulness are not the only suitable factors to determine technology adoption. For particular settings, using different variables from other information technology acceptance models could enhance the specificity and explanatory power of the extended models [30]. For this reason, instead of using the original TAM, or its well-known extensions such as the Technology Acceptance Model 2 (TAM2) [33,34] and the Unified Theory of Acceptance and Use of Technology (UTAUT), we adopted the most relevant and complementary external factors to extend TAM and explain user adoption of diet apps [35].

Perceived usefulness is one of the main factors determining, and main indicator of, behaviors of any kind of technology usage [29,30]. Davis defined *perceived usefulness* as “the degree to which a person believes that using a particular system would enhance his or her job performance” [29]; in this study, we integrated and adapted this definition of perceived usefulness—the degree to which a person believes that using a particular system would enhance their health and well-being to make it more user-centric. Our second construct was *perceived ease of use*, which is described by Davis as ”the degree to which a person believes that using a particular system would be free from effort [29],“ and we wholly integrated this definition into our model.

The major goal of the TAM framework is to understand the behavioral intention of users (acceptance) toward and their actual use of a technology [36,37]. Davis suggested that future technology acceptance studies should focus on other principles that influence usefulness, ease of use, and user acceptance [37]. Thus, price-value, trust, and perceived risk factors were used to extend the TAM framework in this study. These factors are crucial to exploring the perspective of users in the mHealth domain [3,38,39].

The *price-value* factor is related to the subscription fees of the service offered by the mobile health services. It has a direct effect on user acceptance; therefore, we adopted price-value as an external factor. We investigated the direct relationship of user satisfaction on the price that the users pay and whether the service matched their needs [38,40,41]. Moreover, *trust* is a powerful external factor since trust plays a major role in attracting new users and retaining existing users. If a person believes in the service administered by an mHealth app, they are likely to adopt the service. According to the TAM framework, a person’s beliefs can encourage their assertiveness and intention to adopt the technology [3,42,43]. Hence, we integrated trust as another external factor in this study.

**Figure 1:**
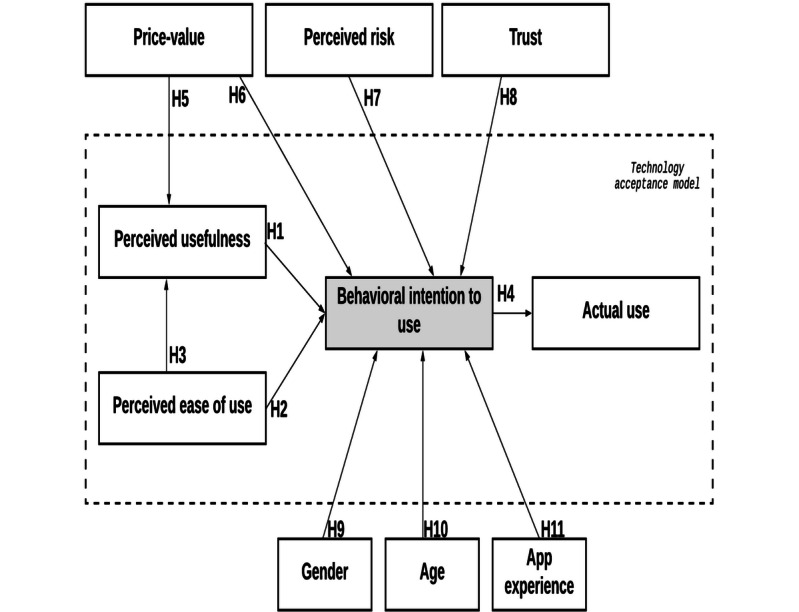
Theoretical framework.

The external factor *perceived risk* can be defined as a “person’s perception of uncertainty in the use of mHealth services and its severity in terms of consequences [39].” There are some risks posed by mHealth apps, such as privacy invasion, legal, and performance risks [44]. Researchers have shown that privacy and perceived risk can predict user adoption [45,46], hence we used perceived risk in our extended model. Finally, some control variables were added to enhance the explanatory potential of the study. Gender, age, and previous mHealth app experience were selected as covariates. Considering these factors, hypotheses and the research framework were established; they are illustrated in Figure 1 and are summarized in Table 1.

**Table 1:** Hypotheses list.

Label	Hypotheses
H1	Perceived usefulness positively affects the intention to use the app.
H2	Perceived ease of use positively affects the intention to use the app.
H3	Perceived ease of use positively affects the perceived usefulness of the app.
H4	Behavioral intention to use positively affects the actual use of the app.
H5	Price-value factor positively affects the perceived usefulness.
H6	Price-value factor positively affects the intention to use the app.
H7	Perceived risk is negatively associated with one’s adoption intention toward the app.
H8	Trust positively affects the intention to use the app.
H9	Gender difference plays a significant role in the intention to use the app.
H10	Age has a significant role in the intention to use the app.
H11	Previous mHealth app use experience of users has a significant role in the intention to use the app.

## Methods

### Study Design

Multiple-itemed scales were used to quantify each construct. All questions were carefully selected and adapted from the questionnaires used in previous studies [1,3,29,43,47-50]. We adjusted most items to make them more suitable for an mHealth context (please see Multimedia Appendix 1 for references for each item). Perceived usefulness was measured with 4 items (PU1-PU4), perceived ease of use was measured with 4 items (PEOU1-PEOU4), behavioral intention to use was measured with 5 items (BI1-BI5), price-value was measured with 3 items (PV1-PV3), perceived risk was measured with 6 items (PR1-PR6), and trust was measured with 3 items (T1-T3).

All of these items were rated by users in a 32-question online survey using a 5-point Likert scale (1, strongly disagree; 3, indecisive; 5, strongly agree). Questions were shuffled and one attention check (trap) question was added to eliminate bias responses. The aim of the attention check question was to ensure that the users were focused. One of the perceived usefulness items was stated as the exact opposite of another perceived usefulness item. In this way, we attempted to minimize the risk of collecting less reliable and inaccurate data from users who were not being attentive [51]. Demographic questions were asked at the beginning of the survey: gender, age group, education level, usage frequency of the app (which directly answers the actual use factor), previous mobile-health app experience, main aim for the use of the app (users were allowed to choose more than one answer from the pool), and membership type (free 1-month standard membership, 3-month standard membership, premium-dietitian service). The survey was prepared in the Turkish language, which is the native language of the app and its users. The survey was sent to Diyetkolik managers to be placed on their survey platform.

### Data Collection

Data were collected with an online survey method on a Typeform web-based platform [52]. This platform is used by Diyetkolik to gather feedback from its users. The survey was distributed randomly to active users who subscribed to Diyetkolik’s mailing lists. Out of 100,000 active users, 50,000 users who checked their weekly emails from Diyetkolik, randomly received the survey link via email. A brief explanation of the project was given in the email and indicated that participation in the survey was voluntary and completely anonymous (please see Multimedia Appendix 1 for the English version and Multimedia Appendix 2 for the Turkish version of the survey).

The data collection phase took 4 weeks between June 11, 2019 and July 10, 2019. Responses were collected from 840 Diyetkolik users. In order to increase the reliability and accuracy, surveys that had missing entries and inattentive responses were eliminated. Hence, the data count was reduced to 658 from the original 840.

### Statistical Procedure

Data were analyzed using SmartPLS 3 (SmartPLS GmbH) and SPSS (version 24.0; IBM Corp) software. Data were gathered in an Excel (Microsoft Inc) file and the shuffled items were ordered categorically. Partial least square, a component-based structural equation modeling technique, was used to examine the research model and test the first 8 hypotheses. Partial least square methods are particularly convenient for complex and large research models to test both reflective and formative constructs. Hence, partial least square-based structural equation modeling was better suited to this research than covariance-based structural equation modeling techniques [39]. For the last 3 hypotheses and for demographics, analysis of variance (ANOVA) was used (at the significance levels *P*<.01 and *P*<.05).

### Analysis of the Measurement Model in Structural Equation Modeling

To assess construct validity and internal accuracy, we used discriminant and convergent validity measures that complemented each other [1]. Cronbach α and composite reliability measures must be around 0.70 for each factor to have a valid internal consistency. Additionally, the average variance extracted and component loadings should be more than 0.50 to have acceptable convergent and construct validity [53].

Calculating the standardized outer loadings of main variables can explain individual variance level, convergent validity, and individual manifest reliability. Thus, items that have more than 0.70 outer loadings count as highly satisfactory. Items which have a value of 0.50 or below should be eliminated to increase the composite reliability. Items valued between 0.40 to 0.70 should be reviewed before dropping out [54].

Composite reliability and Cronbach α values play an important role in analyzing internal consistency. A common rule of thumb indicates that values 0.6 or higher are adequate for exploratory purposes [55].

### Discriminant Validity Measurement

Discriminant validity was checked using the Fornell-Larcker criterion (correlation analysis). According to this criterion, an average variance extracted value above 0.50 is acceptable, and an average variance extracted value above 0.70 shows significant validity.

Another method to check validity is by analyzing the *R*^2^ values. It is a goodness-of-fit measure for linear regression models. It explains the estimated variance of the construct’s relationship with independent variables and higher *R*^2^ values show significantly better model fits [56]. The value of *R*^2^ of underlying variables should be greater than 0.26 [57].

### Hypothesis Testing

To identify the relationships between the variables in the structural model, path coefficient (β) and *t* values were tested by bootstrapping with 1000 subsamples. The relationships among factors are statistically proven with *P* values lower than .01 and with *t* values larger than 1.96.

## Results

### Demographic Characteristics

The demographic characteristics of respondents (n=658) are presented in Table 2. The majority of the respondents were female (438/658, 66.6%) and between the ages of 18-41 years (496/658, 75.4%). Most of the respondents had attained a bachelor’s degree or higher (362/658, 55.0%). Two-thirds of the respondents did not disclose their membership type, and almost one-third of the respondents disclosed that their membership type was basic (211/658, 32.1%).

### Analysis of the Measurement Model in Structural Equation Modeling

In the first round, the items BI5 (0.403), PV3 (0.594), PR1 (–0.772), PR2 (0.330), PR4 (0.556), and PR6 (0.450) were removed from our partial least square structural equation model. Only the items that had outer loadings greater than 0.6 remained, and the final data analysis table was constructed.

Table 3 shows the final measurement model with the mean values. According to the results, internal consistency is satisfied; perceived risk, price-value, and trust (the 3 external variables) had mean values of approximately 3 indicating that the average respondent was indecisive about perceived risk, trust, and price-value.

### Discriminant Validity Measurement

The model passed the validity test with satisfactory results. All variables in Table 4 show discriminant validity with cross loadings above 0.70 (actual use: 1; behavioral intention: 0.732; perceived ease of use: 0.739; price-value: 0.796; perceived risk: 0.873; perceived usefulness: 0.789; trust: 0.873).

For this study, 2 out of 3 *R*^2^ values satisfied the criteria (behavioral intention: *R*^2^=0.635; perceived usefulness: *R*^2^=0.434; perceived ease of use: *R*^2^<0.26).

**Table 2:** Demographics of sample data.

Variable		Participants (n=658), n (%)	
**Gender**			
	Male		163 (24.8)
	Female		438 (66.6)
	Prefer not to say		57 (8.7)
**Age (years)**			
	18-25		169 (25.7)
	26-33		168 (25.5)
	34-41		159 (24.1)
	42-49		112 (17.0)
	50-59		34 (5.2)
	60 +		16 (2.4)
**Educational qualifications**			
	Primary school graduate		9 (1.4)
	High school graduate		91 (13.8)
	Bachelor’s degree		247 (37.5)
	University student		102 (15.5)
	Post-graduate degree and higher		115 (17.5)
	2-year degree graduate		94 (14.3)
**App experience**			
	Yes		314 (47.7)
	No		344 (52.3)
**Membership type**			
	1-month standard subscription		5 (0.8)
	3-month standard subscription		2 (0.3)
	Premium service		1 (0.2)
	Basic (free) membership		211 (32.1)
	Prefer not to say		446 (67.8)

**Table 3:** The measurement model with mean values.

Factors		Loadings		Mean		Average variance extracted	Composite reliability		Cronbach α	
**Perceived usefulness**						0.623	0.868		.798	
	PU1	0.710		3.69						
	PU2	0.806		3.73						
	PU3	0.774		3.89						
	PU4	0.859		3.70						
**Perceived ease of use**						0.546	0.780		.580	
	PEOU1	0.631		4.05						
	PEOU3	0.695		3.28						
	PEOU4	0.870		3.59						
**Price-value**						0.634	0.772		.450	
	PV1	0.905		3.12						
	PV2	0.669	3.05							
**Perceived risk**					0.762			0.865		.688
	PR3	0.884	2.84							
	PR5	0.861	3.03							
**Trust**					0.762			0.906		.884
	T1	0.852	3.33							
	T2	0.854	3.22							
	T3	0.912	3.36							
**Behavioral intention**					0.535			0.820		.708
	BI1	0.678	3.40							
	BI2	0.633	2.55							
	BI3	0.752	3.53							
	BI4	0.846	3.28							

**Table 4:** Correlation matrix of the square root of the average variance extracted for discriminant validity.

Variable	Variable						
	Actual use	Behavioral intention	Perceived ease of use	Price-value	Perceived risk	Perceived usefulness	Trust
Actual use	1	0.322	0.312	0.21	–0.218	0.332	0.117
Behavioral intention	0.322	0.732	0.697	0.684	–0.248	0.61	0.583
Perceived ease of use	0.312	0.697	0.739	0.647	–0.254	0.641	0.481
Price-value	0.21	0.684	0.647	0.796	–0.202	0.536	0.48
Perceived risk	–0.218	–0.248	–0.254	–0.202	0.873	–0.366	–0.247
Perceived usefulness	0.332	0.61	0.641	0.536	–0.366	0.789	0.462
Trust	0.117	0.583	0.481	0.48	–0.247	0.462	0.873

### Hypothesis Testing

The results of partial least square model for the first 8 hypotheses of the study are illustrated in Table 5. Perceived risk’s direct effect on behavioral intention (β=0, *t*=0.01, *P*=.99) was not accepted. The direct effects of perceived ease of use on perceived usefulness (β=0.506, *t*=12.98, *P*<.001), perceived ease of use on behavioral intention (β=0.293, *t*=6.85, *P*<.001), behavioral intention on actual use (β=0.322, *t*=9.10, *P*<.001), and price-value on behavioral intention (β=0.303, *t*=7.67, *P*<.001) were significant.

**Table 5:** Results of partial least square for H1 to H8.

Hypothesis	Path	β	*t* value	*P* value	Result
H1	Perceived usefulness→behavioral intention	0.156	4.28	<.001	Accepted
H2	Perceived ease of use→behavioral intention	0.293	6.85	<.001	Accepted
H3	Perceived ease of use→perceived usefulness	0.506	12.98	<.001	Accepted
H4	Behavioral intention→actual use	0.322	9.10	<.001	Accepted
H5	Price-value→perceived usefulness	0.209	5.03	<.001	Accepted
H6	Price-value→behavioral intention	0.303	7.67	<.001	Accepted
H7	Perceived risk→behavioral intention	0	0.01	.99	Not accepted
H8	Trust→behavioral intention	0.225	7.09	<.001	Accepted

Gender had no statistically significant role on the behavioral intention to use the app (mean 3.21, SD 0.873; *F*_1,599_=0 *P*=.98. In addition, age groups showed no association between age and behavioral intention (mean 3.19, SD 0.872; *F*_5,652_=1.931, *P*=.09). Finally, previous app use was not significant (mean 3.19, SD 0.872; *F*_1,656_=2.137, *P*=.14). Hence, H9, H10, and H11 were all rejected (Multimedia Appendix 3).

## Discussion

### General

Considering the vast influence of information and communication technologies in our daily lives, this research aimed to extend TAM framework and to understand the main factors that influenced Diyetkolik users’ acceptance of mobile dietitian services. Identified factors were tested for the variables' hypothesized positive associations with user acceptance of the diet app. The findings showed that perceived ease of use, perceived usefulness, price-value, and trust had a positive impact on the app acceptance at the behavioral level. We only demonstrated that high behavioral intention of users translated into higher usage frequency of the app (actual use). Previous studies [21,29,33,56,58-62] on TAM had also shown similar results. Our study's results were consistent with Ajzen and Fishbein’s theory [62,63] and also in congruence with findings [29] that suggested that behavioral intention was a good predictor of actual technology use. However, perceived risk did not show any significant relationship with behavioral intention to use (*P*=.99). Users used mHealth apps and benefit from the services if they perceived the app to be useful, easy to control, and trustworthy. Moreover, the analysis of age groups and previous app use data showed that there were no statistically meaningful differences (age: *P*=.09; use: *P*=.14) in behavioral intention to use. Our results also revealed there was no major role of gender in the behavioral intention to use (*P*=.98). This conflicts with findings from another study [64] that showed that men perceived mobile diet apps to be more beneficial in managing their lifestyle and eating habits.

We predict that most respondents who did not respond to the membership question had basic membership, because the marketing and sales managers of Diyetkolik have stated that the majority of users own free accounts. Disclosing the subscription type will be made compulsory in any future research, because it could positively affect our analysis to identify the respondent type clearly. By doing this, we can differentiate our questions for different segmentations instead of focusing on all respondents as a homogenous population.

The findings from the survey’s price-value questions suggested that users were ambivalent about the benefits of premium membership (the subscription type that provides dietitian services to the users). Having dietitian services straight from a mobile device, instead of a physical consultation, seems eminently practical for some respondents, but other respondents were indecisive about consulting a dietitian online through the platform. Most users have vigorously opposed or were hesitant about purchasing health services online. Some users were also indecisive about the reliability of the information that dietitians provide on the Diyetkolik platform. These might stem from issues in trust concerning the accreditation and the authenticity of the platform’s dietitians.

Two external factors (trust and price-value) that were used in this study in addition to the TAM factors (perceived ease of use and perceived usefulness) improved our understanding of user acceptance of an mHealth app and showed relevance with behavioral intention to use. The third external factor (perceived risk) did not provide any explanation regarding intention to use, but it can be divided into further categories (legal concerns, privacy risks, etc), and its statistical relationship with behavioral intention can be retested in future research. Therefore, based on our findings, we propose an extended TAM framework.

### Limitations

This study had some limitations. It was conducted in one country (Turkey), and the findings might not be generalizable to related apps in European Union countries or in North America. Demographic variables such as sociocultural differences with regard to diet would create differences between different countries. Future research could study different country settings and compare cross-country data. Although the investigation had a good representative sample, it was conducted online which can cause some problems regarding the attention of respondents. We tried to minimize this effect to a degree by including a trap question.

Other data collection methods might be considered for future studies, for example, inviting previous or occasionally active Diyetkolik users to take part in the study. The insights from former users could be important to understanding the factors behind technology abandonment, resistance, or rejection; there may be close links with behavioral intention to use.

Due to the number of users preferring not to disclose their type of membership, we were unable to identify additional insights from comparing user perceptions about different subscriptions. Future research can leverage digital trace data of user actions on the mobile app and can use data analytics to examine further hypotheses.

### Conclusion

Measuring the user acceptance of mHealth by using an extended TAM model was the primary aim of this study. Our research showed that the perceived ease of use and the perceived usefulness of an mHealth app were associated with the behavioral intention of users to adopt and continue using the app. A mHealth-specific extended TAM model that was developed showed that price-value and trust factors directly affected the behavioral intention to use the app. However, perceived risk and behavioral intention were not associated, and gender, age groups, and previous app use experience had no direct or moderating roles on the intention to use the app.

Despite the aforementioned limitations, the results and findings of the study contribute to mHealth app design and the development of dietary and nutrition mHealth apps. The success of apps is highly dependent on the user’s willingness to adopt them. On a practical level, the insights from this research can benefit nutrition and diet app developers and managers to increase the adoption and actual use of apps. Furthermore, the study proposes a theoretical extension to the literature. TAM is a generic framework which is often used by researchers to study the user acceptance of technologies, however, there are only a few studies [22,44,65] that have explored diet-based mHealth apps. Hence, our expanded TAM framework lays out additional constructs and contributes to technology acceptance literature. We believe that further research will use this model and possibly identify new factors that could be useful in studying user acceptance of diet apps.

As a recommendation, managers might find it useful to focus on promoting the perceived usefulness of the service in order to increase the uptake of premium services by the users. The average respondent in our Diyetkolik case study was hesitant about purchasing health services online, based on the app’s price-value. If the benefits of the premium packages are communicated to the users in innovative ways, and if the users are frequently reassured about the security of the app and the authenticity of the platform’s experts (eg, Diyetkolik’s dietitians), then they might feel more inclined to purchase premium services. This recommendation can be generalizable to other mHealth apps where the user concerns are similar.

## Appendix

Multimedia Appendix 1 English version of survey questions.

Multimedia Appendix 2 Turkish version of survey questions.

Multimedia Appendix 3 Hypotheses 9, 10, and 11 one-way ANOVA results.

## Abbreviations

ANOVA: analysis of variance
mHealth: mobile health
TAM: Technology Acceptance Model
UTAUT: Unified Theory of Acceptance and Use of Technology
