# Supplementary material for: Adoption of Mobile Health Apps in Dietetic Practice: Case Study of Diyetkolik
Source: JMIR Mhealth Uhealth. 2020 Oct 2;8(10):e16911. doi: 10.2196/16911 (PMC7568214; doi:10.2196/16911)
Supplement: Multimedia Appendix 1 [file mhealth_v8i10e16911_app1.pdf]

Survey Questions in English and Sources

	Strongly Disagree	Disagree	Indecisive	Agree	Strongly Agree	Source/s
Perceived Usefulness						
Tracking my health by using an app is useful for me.						Adapted from (Cilliers, Viljoen, & Chinyamurindi, 2017; Davis, 1989)
Using Diyetkolik app on my mobile device to get information on healthy eating is beneficial for me.						Adapted from (Cilliers et al., 2017)
Getting health services on mobile devices is unnecessary.						By authors (attention question)
I think using Diyetkolik mHealth app is useful.						Adapted from (Cilliers et al., 2017; Davis, 1989; Hoque & Sorwar, 2017)
Perceived Ease of Use						
Learning how to use Diyetkolik app is easy.						Adapted from (Cilliers et al., 2017)
I expect that my interaction with Diyetkolik to access diet information would be clear and understandable.						Adapted from (Cilliers et al., 2017; Davis, 1989)
I think online diet consultation is much easier than face-to-face consultation.						Adapted from (Deng, Hong, Ren, Zhang, & Xiang, 2018)

Survey Questions in English and Sources

I think Diyetkolik app's user interface is easy to use.						Adapted from (Deng et al., 2018)
	**Strongly Disagree**	**Disagree**	**Indecisive**	**Agree**	**Strongly Agree**	
Intention to use						
I am willing to use Diyetkolik app and their services to reach diet plans etc.						Adapted from (Deng et al., 2018)
I intend to learn more about using Diyetkolik services.						Adapted from (Hoque & Sorwar, 2017)
I intent to use Diyetkolik app every day.						Adapted from (Deng et al., 2018)
I am going to recommend Diyetkolik app to my family and friends.						Adapted from (Chong, 2013; Kim, Ferrin, & Rao, 2008)
I am not planning to use any Diyetkolik alternative app on the next 6 months ahead.						By authors
Perceived Risk						
I believe my private data is well protected by Diyetkolik.						Adapted from (Hoque & Sorwar, 2017)
I am worried that Diyetkolik might share my personal information with the third parties.						Adapted from (Chen, Han, & Yu, 1996; Kim et al., 2008)

Survey Questions in English and Sources

I am worried that using Diyetkolik app will not satisfy my needs to keep myself healthy.						Adapted from (Deng et al., 2018)
Compared to face-to-face consultation, I am worried about the efficiency of Diyetkolik's online diet consultation service.						Adapted from (Deng et al., 2018)
I have doubts on the services provided by Diyetkolik will not meet my expectations.						Adapted from (Deng et al., 2018)
I am worried that my credit/debit card details might get stolen while I am paying my subscription fee.						Adapted from (Chong, 2013; Kim et al., 2008)
	**Strongly Disagree**	**Disagree**	**Indecisive**	**Agree**	**Strongly Agree**	
Trust						
Generally, I think dietitians on Diyetkolik service platform are trustworthy.						Adapted from (Deng et al., 2018)
Dietitians on Diyetkolik platform are verified, and I think their credibility is guaranteed by the firm.						Adapted from (Deng et al., 2018)
In general, I trust health advices/tips given by dietitians on the Diyetkolik platform.						Adapted from (Deng et al., 2018)

Survey Questions in English and Sources

	Strongly Disagree	Disagree	Indecisive	Agree	Strongly Agree	
Price-value						
Advantages offered by Diyetkolik services for the subscribed (paid) members can provide more benefits to my health.						Adapted from (Venkatesh, Thong, & Xu, 2012)
Premium membership package 'Dietitian Service' can help me to save time by providing same service online. Hence, I get similar service with less effort and for a fair price.						Adapted from (Venkatesh et al., 2012)
I believe subscription fees of Diyetkolik is affordable, and fair compared to service provided by them.						Adapted from (Venkatesh et al., 2012)

DEMOGRAPHIC QUESTIONS

1) Gender: Male ( ) Female ( )

2) Age:
18-25 ( ) 26-33 ( )
34-41 ( ) 42-49 ( )
50-59 ( ) 60+( )

3) Education level:
Primary school degree ( )
High school degree ( )
Bachelor's degree ( )
University student ( )
Post-graduate degree and higher ( )
2-year degree graduate ( )

4) Previous diet app usage experience: Yes ( )
No ( )

5) Do you use Diyetkolik app?

No, I did not download it on my phone. ( )
No, I downloaded the app, but I do not use it. ( )
Yes, I use it rarely. ( )
Yes, I use it regularly. ( )

6) What is your membership type?

Free/basic user ( )
Standard membership (1month) ( )
Standard membership (3months) ( )
Dietitian service (Premium) ( )

7) What is your aim to use this app? (choose as many as you like)

Following the most appropriate diet plan due to my illness (obesity, diabetes etc.)
Healthy weight loss
Healthy weight gain
Maintaining weight.
Keeping fit.
Getting reliable information on healthy lifestyle.
Calorie tracking.
Fitness/exercise tracking
Water intake tracking.
Finding food recipes.
Learning healthy eating habits with the help of dietitian/s
Other
