# Supplementary material for: Adoption of Mobile Health Apps in Dietetic Practice: Case Study of Diyetkolik
Source: JMIR Mhealth Uhealth. 2020 Oct 2;8(10):e16911. doi: 10.2196/16911 (PMC7568214; doi:10.2196/16911)
Supplement: Multimedia Appendix 2 [file mhealth_v8i10e16911_app2.pdf]

Sample screenshots of the Survey Instrument in Turkish

DİYETKOLİK e-ANKET ÇALIŞMASI

Sağlayacağınız değerli geri bildirimler çerçevesinde mobil uygulamamızın sizler tarafından kabulü ve kullanımı üzerindeki önemli faktörleri ortaya çıkarmak istiyoruz!

Bu bilimsel çalışma anonim olup, Kadir Has Üniversitesi, Yönetim Bilişim Sistemleri Departmanı tarafından analiz edilecektir. Veriler üçüncü taraflar ile paylaşılmayacaktır.

Katılımınız bizler için çok önemlidir. Sağlıklı günler dileriz!

Start press ENTER

1→ Cinsiyetiniz

- A Kadın
- B Erkek

2→ Yaşınız

- A 18-25
- B 26-33
- C 34-41
- D 42-49
- E 50-59
- F 60 ve üzeri

3→ Eğitim durumunuz

- A Eğitimim yok
- B İlköğretim mezunu
- C Lise mezunu
- D Yüksek okul mezunu
- E Lisans öğrencisi
- F Lisans mezunu
- G Yüksek lisans ve üstü

4→ Daha önce Diyetkolik'e benzer herhangi bir sağlık uygulaması kullandınız mı?

- A Evet
- B Hayır

5→ Diyetkolik'in mobil uygulamasını kullanıyor musunuz?

- A Hayır, uygulamayı telefonuma indirmedim.
- B Hayır, telefonuma indirdim fakat kullanmıyorum.
- C Nadiren kullanıyorum.
- D Evet, uygulamayı düzenli kullanıyorum.

6→ Uygulamayı kullanmanızdaki amacınız hangisi ya da hangileridir?

Choose as many as you like

- A Hastalığıma uygun beslenmek (obezite, diyabet vb.)
- B Sağlıklı bir şekilde kilo vermek
- C Sağlıklı bir şekilde kilo almak
- D Kilomu korumak
- E Fit bir vücuda sahip olmak
- F Diyet ve egzersiz konularında güvenilir bilgilere ulaşmak
- G Kalori takibi yapmak
- H Egzersiz takibi yapmak
- I Su takibi yapmak
- J Yemek tarifleri öğrenmek
- K Diyetisyen kontrolünde sağlıklı beslenme alışkanlığı edinmek
- L Other

7→ Üyelik tipiniz nedir?

- [A] Ücretsiz üye
- [B] 1 aylık Standart Paket üyesi (25 TL)
- [C] 3 aylık Standart Paket üyesi (57 TL)
- [D] Diyetisyen Hizmeti (133 TL'den başlayan fiyatlar)

" Bu bölümde yer alan cümleleri aşağıdaki ölçütlere göre 1 ile 5 arasında puanlayınız.
*Kesinlikle Katılmıyorum/Kesinlikle Hayır [1]
*Katılmıyorum/Hayır [2]
*Kararsızım [3]
*Katılıyorum/Evet [4]
*Kesinlikle Katılıyorum/Kesinlikle Evet [5]

Continue press ENTER

8→ Sağlığımı mobil bir uygulama üzerinden takip etmek benim için yararlıdır.

☆ ☆ ☆ ☆ ☆
1 2 3 4 5

9→ Mobil cihazım üzerinden Diyetkolik uygulamasını kullanarak sağlıklı beslenmeyle ilgili bilgilere ulaşmak benim için faydalıdır.

☆ ☆ ☆ ☆ ☆
1 2 3 4 5

10→ Mobil cihazım üzerinden Diyetkolik uygulamasını kullanarak sağlık hizmetleri almak gereksizdir.

☆ ☆ ☆ ☆ ☆
1 2 3 4 5

11→ Paket fiyatlandırmalarının verilen hizmetlere göre uygun olduğu kanısındayım.

☆ ☆ ☆ ☆ ☆
1 2 3 4 5

12→ Bana göre, Diyetkolik mobil sağlık uygulaması yararlı bir uygulamadır.

☆ ☆ ☆ ☆ ☆
1 2 3 4 5

13→ Diyetkolik uygulamasının kullanımını öğrenmek zor degil.

☆ ☆ ☆ ☆ ☆
1 2 3 4 5

14→ Diyetkolik uygulamasını kullanırken diyet ve egzersiz bilgilerine ulaşmanın daha anlaşılır ve net olması gerektiği yönünde bir beklentim var.

☆ ☆ ☆ ☆ ☆
1 2 3 4 5

15→ En üst paket olan "Diyetisyen Hizmeti" beni sürekli kliniğe gitmekten kurtaracağı için zaman tasarrufu sağlar ve yine aynı hizmeti daha az eforla uygun bir fiyata almış olurum.

☆ ☆ ☆ ☆ ☆
1 2 3 4 5

16→ Bence internet ortamından (uzaktan) diyetisyenlik hizmeti almak ve diyet bilgilerine ulaşmak kliniğe gitmekten daha pratiktir.

☆ ☆ ☆ ☆ ☆
1 2 3 4 5

17→ Diyetkolik uygulamasını sağlıklı yaşama ilişkin hizmetlere ulaşabilmek için kullanmaya istekliyim.

☆ ☆ ☆ ☆ ☆
1 2 3 4 5

18→ Diyetkolik uygulaması ve hizmetlerinin kullanımı hakkında daha fazla bilgi edinmek isterim.

☆ ☆ ☆ ☆ ☆
1 2 3 4 5

19→ Uygulamanın sunduğu özelliklere bakıldığında ücretli üyelik sağlığım açısından bana daha fazla katkı sağlar.

☆ ☆ ☆ ☆ ☆
1 2 3 4 5

20→ Günlük hayatımda düzenli bir şekilde bu uygulamayı ve hizmetlerini kullanmaya niyetim var.

☆ ☆ ☆ ☆ ☆
1 2 3 4 5

21→ Diyetkolik uygulamasının kullanıcı arayüzünü açık ve kolay anlaşılır buluyorum.

☆ ☆ ☆ ☆ ☆
1 2 3 4 5

22→ Arkadaşlarıma/aileme Diyetkolik uygulamasını kullanmalarını öneririm.

☆ ☆ ☆ ☆ ☆
1 2 3 4 5

23→ Diyetkolik uygulamasının kişisel verilerimi üçüncü şahıslarla paylaşma ihtimali beni endişelendiriyor.

☆ ☆ ☆ ☆ ☆
1 2 3 4 5

24→ Önümüzdeki 6 ay içerisinde Diyetkolik'e alternatif bir uygulama kullanmayı planlamıyorum.

☆ ☆ ☆ ☆ ☆
1 2 3 4 5

25→ Bence Diyetkolik'le paylaştığımız kişisel verilerimiz güvenli bir şekilde korunmaktadır.

☆ ☆ ☆ ☆ ☆
1 2 3 4 5

26→ Diyetkolik uygulamasını kullanmamın sağlığımı koruma konusunda beni yeterince tatmin etmeyeceği yönünde kuşkularım var.

☆ ☆ ☆ ☆ ☆
1 2 3 4 5

27→ Diyetkolik platformunda hizmet veren diyetisyenlerin genel olarak güvenilir kişiler olduklarını düşünüyorum.

☆ ☆ ☆ ☆ ☆
1 2 3 4 5

28→ Klinik diyetisyen hizmeti ile karşılaştırıldığında internet üzerinden sağlanan bu hizmetin yeterliliği bende endişe yaratıyor.

☆ ☆ ☆ ☆ ☆
1 2 3 4 5

29→ Uygulamanın sunduğu hizmetlerin beklentilerimi karşılamayacağı konusunda şüphelerim var.

☆ ☆ ☆ ☆ ☆
1 2 3 4 5

30→ Üyelik ödemesi yapacağım zaman kredi kartı bilgilerimin çalınabileceği düşüncesi beni endişelendiriyor.

☆ ☆ ☆ ☆ ☆
1 2 3 4 5

31→ Bu platformdaki diyetisyenlerin onaylı kişiler olduklarının garantisinin verildiğini düşünüyorum.

☆ ☆ ☆ ☆ ☆
1 2 3 4 5

32→ Bu platformdaki diyetisyenlerin verdikleri bilgilerin doğruluğuna güveniyorum.

☆ ☆ ☆ ☆ ☆
1 2 3 4 5
