# Supplementary material for: Adoption of Mobile Health Apps in Dietetic Practice: Case Study of Diyetkolik
Source: JMIR Mhealth Uhealth. 2020 Oct 2;8(10):e16911. doi: 10.2196/16911 (PMC7568214; doi:10.2196/16911)
Supplement: Multimedia Appendix 3 [file mhealth_v8i10e16911_app3.pdf]

Statistics for Demographic variables

Gender

Descriptive

meanBI

	N	Mean	Std. Deviation	Std. Error	95% Confidence Interval for Mean Lower Bound	95% Confidence Interval for Mean Upper Bound	Minimum	Maximum
MALE	163	3.21	.891	.070	3.07	3.34	1	5
FEMALE	438	3.21	.867	.041	3.13	3.29	1	5
Total	601	3.21	.873	.036	3.14	3.28	1	5

ANOVA

meanBI

	Sum of Squares	df	Mean Square	F	Sig.
Between Groups	.000	1	.000	.000	.983
Within Groups	457.250	599	.763		
Total	457.250	600			

Prefer not to say answers removed.

Age

Descriptive

meanBI

	N	Mean	Std. Deviation	Std. Error	95% Confidence Interval for Mean Lower Bound	95% Confidence Interval for Mean Upper Bound	Minimum	Maximum
18-25	169	3.06	.946	.073	2.92	3.21	1	5
26-33	168	3.29	.851	.066	3.16	3.42	1	5
34-41	159	3.15	.786	.062	3.03	3.27	1	5
42-49	112	3.32	.846	.080	3.16	3.48	1	5
50-59	34	3.07	1.054	.181	2.70	3.43	1	5
60+	16	3.11	.677	.169	2.75	3.47	2	4
Total	658	3.19	.872	.034	3.12	3.25	1	5

ANOVA

meanBI

	Sum of Squares	df	Mean Square	F	Sig.
Between Groups	7.285	5	1.457	1.931	.087
Within Groups	492.036	652	.755		
Total	499.320	657			

Previous app experience

Descriptive

meanBI

	N	Mean	Std. Deviation	Std. Error	95% Confidence Interval for Mean Lower Bound	95% Confidence Interval for Mean Upper Bound	Minimum	Maximum
Yes	314	3.24	.893	.050	3.14	3.34	1	5
No	344	3.14	.851	.046	3.05	3.23	1	5
Total	658	3.19	.872	.034	3.12	3.25	1	5

ANOVA

meanBI

	Sum of Squares	df	Mean Square	F	Sig.
Between Groups	1.622	1	1.622	2.137	.144
Within Groups	497.699	656	.759		
Total	499.320	657			
